# Prolonged grief and insomnia symptoms in cancer-bereaved parents: a latent class analysis

**DOI:** 10.1186/s12888-026-07955-9

**Published:** 2026-03-13

**Authors:** Josefin Sveen, Maarten C. Eisma, Lilian Pohlkamp, Ulrika Kreicbergs, Lonneke I. M. Lenferink

**Affiliations:** 1 Department of Women’s and Children’s Health, Uppsala University, Uppsala University Hospital, Uppsala, 751 85 Sweden; 2 Department of Clinical Psychology and Experimental Psychopathology, Faculty of Behavioral and Social Sciences, University of Groningen, Groningen, The Netherlands; 3 Department of Health Care Sciences, Marie Cederschiöld University, Stockholm, Sweden; 4 Great Ormond Street Institute of Child Health, University College London, London, UK; 5 Department of Psychology, Health & Technology, Faculty of Behavioural Management and Social Sciences, University of Twente, P.O. Box 217, Enschede, 7500 AE The Netherlands; 6 Department of Clinical Psychology, Faculty of Social Sciences, Utrecht University, Utrecht, The Netherlands

**Keywords:** Prolonged grief disorder, Insomnia, Depression, Coping, Rumination, Latent class analyses

## Abstract

**Background:**

Parents who experience the death of a child may develop a range of mental health problems including insomnia and prolonged grief disorder (PGD). Insomnia symptoms predict worsening of prolonged grief symptoms and persons with more chronic insomnia trajectories may be at risk of developing PGD. However, it remains to be established when prolonged grief and insomnia symptomatology co-occur and which potential risk factors heighten the risk of comorbid insomnia and prolonged grief.

**Methods:**

To address these questions, we conducted a cross-sectional, registry-based survey study involving 225 Swedish parents who lost a child to cancer 1–5 years ago. We assessed sociodemographic and loss-related characteristics, self-rated health, grief rumination, and depressive, posttraumatic stress, prolonged grief, and insomnia symptoms.

**Results:**

A latent class analysis yielded a three-class solution, including a resilient class (*n* = 58, 25.9%), characterized by predominantly low to moderate odds of insomnia and prolonged grief symptoms, a prolonged grief class (*n* = 75, 33.1%), characterized by moderate to high probability of prolonged grief symptoms and low to moderate probability of insomnia symptoms, and a comorbid prolonged grief/insomnia class (*n* = 92, 41.0%), characterized by predominantly high odds of prolonged grief and insomnia symptoms. Univariate analyses of covariates demonstrated that self-rated health, grief rumination, depressive, posttraumatic stress, prolonged grief, and insomnia symptoms differed across classes. The resilient class demonstrated the lowest levels of these covariates, the prolonged grief class higher levels, and the prolonged grief/insomnia class the highest levels. Results for sociodemographic and loss-related variables were less consistent. Multivariate analyses including all covariates simultaneously identified older age and grief rumination variables uniquely distinguishing two or more latent classes.

**Conclusions:**

Results confirm that bereaved parents are at risk of experiencing comorbid insomnia and prolonged grief. However, substantive subgroups of this population report prolonged grief symptoms without severe insomnia symptoms or demonstrate resilience. Older age of the parent and rumination emerged as potential risk factors for co-occurring severe insomnia and prolonged grief symptoms. Rumination may be a key target for interventions for bereaved parents experiencing these mental health problems.

**Clinical trial number:**

Not applicable.

**Supplementary Information:**

The online version contains supplementary material available at 10.1186/s12888-026-07955-9.

## Background

For some, bereavement can precipitate a range of mental health problems, including major depression, posttraumatic stress disorder (PTSD), and severe, persistent, and disabling grief, often termed prolonged grief [1]. Diagnoses characterized by prolonged grief have recently been included in the form of prolonged grief disorder (PGD) in the International Classification of Diseases, eleventh edition (ICD-11) [2]and the Diagnostic and Statistical Manual of Mental Disorders, fifth edition, text revision (DSM-5-TR) [3]. Sleep disturbances are also commonly experienced after bereavement [4, 5] and are elevated among people with prolonged grief, particularly if they experience comorbid depression [6, 7]. Parents bereaved of a child appear at higher risk for loss-related mental health problems [8, 9].

Sleep disturbance is increasingly regarded as a transdiagnostic risk factor [10, 11]. For example, past research has demonstrated that insomnia symptoms increase depressive and posttraumatic stress symptoms over time [10, 12]. Additionally, evidence-based treatments for insomnia, such as cognitive-behaviour therapy for insomnia (CBT-I), have been shown to reduce depressive and posttraumatic stress symptoms [13, 14]. It is theorized that sleep, particularly REM sleep, plays a critical role in the cognitive processing of emotional memories [11, 15, 16]. Accordingly, disruption of healthy sleep could interfere with the processing of negative life-events, such as bereavement [4].

Against this background, researchers have begun to explore in longitudinal studies whether sleep disturbances may disrupt the grieving process, and, relatedly, whether CBT-I may be an effective treatment for persons with comorbid insomnia and PGD. In one longitudinal cohort study, self-reported sleep problems and lower sleep duration before bereavement predicted an increased risk of prolonged grief at six-year follow-up [17]. Another longitudinal survey study found that changes in insomnia symptoms predicted changes in prolonged grief symptoms, but not vice versa, over six-month intervals [18]. Additionally, a longitudinal study applying a growth mixture model demonstrated that insomnia symptoms following bereavement follow three trajectories: chronic insomnia (consistently high insomnia symptoms, 10%), recovering (elevated insomnia symptoms that decline over time, 43%), and resilient (consistently low insomnia symptoms, 47%) [19]. Those with a chronic insomnia trajectory had the highest odds of developing probable PGD. Moreover, changes in insomnia and prolonged grief symptoms over time were strongly positively associated. A small randomized controlled trial further demonstrated that internet-based CBT-I had strong long-term effects on prolonged grief symptoms [20].

In light of these findings, it appears pertinent to identify those bereaved individuals who may be most likely to experience co-occurring prolonged grief and insomnia, as these individuals could potentially benefit most from insomnia treatment. Previous research has typically examined such co-occurrence using cut-off scores on prolonged grief measures, comparing sleep outcomes between individuals with and without probable prolonged grief [21, 22]. However, this binominal approach provides limited information about subpopulations of bereaved persons showing different levels of and combinations of symptomatology types. Latent class analysis (LCA) identifies unobserved subgroups of individuals based on patterns of symptom endorsement. Prior LCA studies in populations exposed to stressful life events have identified distinct symptom profiles that vary in both severity and symptom combinations. For instance, LCA studies in various samples of persons exposed to stressful life events have identified several posttraumatic stress subtypes that differ in terms of severity of symptoms (e.g., classes with low, moderate, and high odds of PTSD symptoms) [23, 24]. Additionally, LCA can distinguish the combination of different subsets of symptoms (e.g., low odds PTSD and dissociation symptoms, high odds of PTSD and dissociation symptoms, and high odds of PTSD symptoms and low odds of dissociation symptoms) [25]. Identifying different classes of persons with particular symptomatology can enhance understanding of symptom presentations in at-risk populations, such as bereaved parents. Establishing correlates of class membership can shed light on factors that may heighten or lower the odds of experiencing particular mental health problems.

### The current study

We aimed to identify symptom-profiles of prolonged grief and insomnia, and correlates thereof, in cancer-bereaved parents. Based on prior LCA research in bereaved persons [26], we expected to find at least three classes. A first class with relatively low probability of endorsement of prolonged grief and insomnia symptoms (i.e., a resilient class). A second class showing high probability of prolonged grief symptoms, but low probability of insomnia symptoms (i.e., a prolonged grief only class). The third class is expected to represent high probability of endorsing prolonged grief and insomnia symptoms (i.e., a prolonged grief/insomnia class).

In light of previous research showing that comorbid depressive symptoms heighten the risk of experiencing sleep disturbances in persons with probable prolonged grief [6, 7] and the established links between posttraumatic stress symptoms and both insomnia and prolonged grief symptoms [12, 27], we expected that the classes would differ on posttraumatic stress and depressive symptom levels. Specifically, we predicted the most severe comorbid symptoms in the prolonged grief/insomnia class, followed by the prolonged grief only class, and the resilient class. Similarly, considering the ample evidence supporting a detrimental role of ruminative coping in prolonged grief and insomnia [28, 29], we expected that grief rumination levels to be higher in classes showing more severe symptomatology levels.

Furthermore, we explored whether the identified classes would differ on potentially relevant background and loss-related characteristics [30, 31]. Specifically, we examined whether classes differed on sex, age, education level, employment status, marital status, time since loss, child age, loss of an only child (vs. not), sickness duration, and relapse status (death after relapse vs. no relapse).

## Methods

### Design

A nationwide cross-sectional registry-based postal survey was conducted in 2016 among parents who had lost a child to cancer 1–5 years ago in Sweden [9].

### Participants and procedures

Parents were identified through the Swedish Childhood Cancer Registry and the Swedish Population Registry. Inclusion criteria were being a parent of a child who was diagnosed with a malignancy before the age of 17 and died of the malignancy before the age of 25, during the period August 2010 to July 2015. The parent had to live in Sweden at the time of the study and understand the Swedish language. A total of 512 parents were eligible and were sent an information letter that explained the purpose and procedure of the study. The letter also provided contact details for the research team should the parents have any further questions. Verbal consent from both the mother and father of each child was obtained separately by phone. Some parents e-mailed the research group consenting to participate after receiving the letter, thus they were not contacted by phone. Of the eligible participants, 373 parents consented to take part in the study and were sent a questionnaire, along with a prepaid return envelope, 76 parents could not be reached, and 63 parents declined participation. Reminder calls were made to participants who did not return the questionnaire within one month. In total, 232 parents (62%) returned the questionnaire. The sample included in the present study consisted of 225 parents who had completed the Prolonged Grief Disorder-13 (PG-13) scale [32].

### Measures

#### Demographic and loss-related variables

The information regarding the child’s cancer diagnosis, sex, age of the child at diagnosis and death, time since death, and duration between diagnosis and death was obtained from the Swedish Childhood Cancer registry. The questionnaire included questions on parents’ sex, age, marital status, employment status, education level (primary/secondary school vs. university), number of children, and the occurrence of relapses (yes/no) in the child.

#### Prolonged Grief Disorder-13 (PG-13)

The PG-13 is a 13-item self-reported measure assessing symptoms of prolonged grief, in line with Prigerson et al. [33] PGD criteria. The PG-13 instrument includes 11 items assessing cognitive, behavioural, and emotional symptoms, such as yearning, emotional pain, avoiding reminders, and trouble accepting the loss. Two items assessing duration and impairment are dichotomous (Yes/No). Four symptom items are rated on a frequency scale from 1 (not at all) to 5 (several times a day), and the other six symptom items are rated on an intensity scale from 1 (not at all) to 5 (overwhelmingly). The total score ranges from 11 to 55. The Swedish version of PG-13 has shown satisfactory psychometric properties [32, 34] and a preliminary cutoff score ≥ 35 indicating probable PGD according to Prigerson’s 2009 criteria has been established [32]. In the current study, the PG-13 showed good internal consistency (α = 0.85).

#### Insomnia Severity Index (ISI)

Insomnia symptoms were assessed using the ISI [35]. The ISI is a self-report instrument that includes seven items that assess the severity of various aspects of insomnia, such as sleep-onset and sleep maintenance difficulties, satisfaction with sleep patterns, interference with daily functioning, noticeability of impairment attributed to the sleep problem, and the degree of concern or distress caused by the sleep problem. The items are rated on a 5-point Likert scale ranging from 0 (no problem) to 4 (severe problem). The total score ranges from 0 to 28, and a cutoff ≥ 10 is used for clinical levels of insomnia. The psychometric properties of ISI are adequate [36]. In the current study, the ISI showed excellent internal consistency (α = 0.90).

#### Utrecht Grief Rumination Scale (UGRS)

Grief rumination, defined as repetitive and recurrent thought about causes and consequences of a loss and negative loss-related emotions, was assessed using the UGRS [37, 38]. Total scores range from 15 to 75, yielding an overall grief rumination score. The scale includes five subscales: Meaning, Relationships, Counterfactuals, Injustice, and Reactions, assessing ruminative thoughts related to the meaning of the loss, others’ reactions, counterfactual thinking, perceived injustice, and emotional responses. Participants were asked to rate how often they have experienced certain thoughts over the past month. The 15-item UGRS is rated on a 5-point Likert scale ranging from 1 (never) to 5 (very often). The Swedish version of UGRS has shown satisfactory psychometric properties [38]. In the current study, the UGRS showed excellent internal consistency (α = 0.92).

#### Posttraumatic Stress Disorder Checklist for DSM-5 (PCL-5)

The self-report measure PCL-5 was used to assess symptoms of posttraumatic stress symptoms [39]. The PCL-5 consists of 20 items, rated on a 5-point Likert scale from 0 (not at all) to 4 (extremely). The items refer to feelings and thoughts during the past month and to a specific event, which in this case is the loss of a child. The checklist consists of 20 items that can be divided into four subscales: Intrusion, Avoidance, Negative alterations in cognitions and mood, and Alterations in arousal and reactivity. The Swedish version has shown satisfactory psychometric properties [40]. In the current study, the PCL-5 showed excellent internal consistency (α = 0.94).

#### Montgomery-Åsberg Depression Rating Scale (MADRS)

To assess symptoms of depression, the self-report measure MADRS was used [41]. MADRS, consisting of 9 items, assesses sadness, inner tension, reduced sleep, reduced appetite, concentration difficulties, fatigue, inability to feel, pessimistic thoughts, and suicidal thoughts. Each item is rated on a 6-point scale, and the total score ranges from 0 to 54, with a higher score indicating a greater risk of depression. In the current study, the MADRS showed excellent internal consistency (α = 0.90).

#### Self-rated health (SRH)

SRH was assessed with the question “How would you rate your general health?” rated on a 5-point Likert scale ranging from 1 (very good) to 5 (very poor).

### Statistical analyses

An LCA was performed using LatentGOLD version 5.0.0 [42]. The LCA included 11 dichotomized indicators for prolonged grief and seven for insomnia. Item responses were dichotomized to distinguish between absent versus clinically relevant symptom endorsement, consistent with the ordinal scaling of the items. Scores of ≥ 3 on the 1–5 scale reflect at least moderate symptom severity and were therefore coded as symptom presence, whereas scores of 1–2 were coded as symptom absence. This threshold is consistent with thresholds used in comparable studies and indicates the presence of a symptom [26]. All persons completed the prolonged grief items. For insomnia symptoms, six participants (2.7%) did not complete any items of the ISI. Missing data on indicators were handled using full information maximum likelihood estimation. The Guidelines for Reporting on Latent Class Analyses (GRoLCA) were followed when reporting the findings [26].

First, a one-class model was estimated, followed by models with increasing number of classes up to a six-class model. The latent class model with the best fit was selected based on fit indices, interpretability of the results, and class sizes. Concerning the latter two: models with meaningful/theoretically interpretable classes were preferred, and models containing classes with relatively small sizes were avoided due to potential computational difficulties in examining possible correlates of class-membership.

We based our model selection on the following fit indices: (i) Akaike’s Information Criterion (AIC), (ii) Bayesian Information Criterion (BIC), and (iii) Sample-Size Adjusted Bayesian Information Criterion (SA-BIC), with lower values indicating better fit. Furthermore, (iv) entropy *R*^2^ values were calculated, with values closer to 1 indicated better class-separation, and (v) the bootstrap likelihood ratio test (BLRt) with a *p*-value < 0.05 was used as an indicator of whether the model under consideration had a significantly better fit compared to a model with one class less [43].

Second, we examined to what extent the classes differed in terms of levels of prolonged grief, insomnia, posttraumatic stress, and depression symptoms, as well as grief rumination and self-rated health, by including these as correlates in the model separately using the three-step-approach. The three-step-approach is implemented in LatentGOLD [44], which takes classification error into account when conducting multinominal logistic regression analyses. Third, background and loss-related characteristics were added as correlates of class-membership to the model separately. Missing data on possible correlates were handled using listwise deletion.

Fourth, the correlates from the previous steps (excluding the sum scores of prolonged grief and insomnia symptoms because the individual items were already included as indicators in the LCA) that were significantly univariately related to class-membership were entered simultaneously in a final multivariate model. For each pairwise comparison, the 95% confidence intervals (CIs) were calculated. When zero was not included in the 95% CI, the difference was considered significant.

## Results

### Participant characteristics

Table 1 presents the characteristics of the 225 participants and 151 deceased children. The majority of the sample (59%) was female. Participants were on average 46 years old. Their children were on average 10 years old when they died. The loss took place 4 years previously on average. The mean insomnia levels were just above the clinical cutoff for clinically relevant insomnia levels.

**Table 1:** Bereaved Parents (*N* = 225) and Deceased Children (*N* = 151) Characteristics

**Parents at the time of the study:**	
** Sociodemographic characteristics**	
Sex = female, *n (%)* (*n* = 225)	133 (59.1)
Age (in years), *M (SD)* (*n* = 225)	46.02 (8.15)
Education = Primary/secondary school, *n (%)* (*n* = 224)	117 (52.2)
Employment = unemployed, *n (%)* (*n* = 224)	34 (15.2)
Marital status = single/living apart together, *n (%)* (*n* = 224)	41 (18.3)
Only child died = yes, *n (%)* (*n* = 224)	23 (10.3)
Years since loss, *M (SD)* (*n* = 225)	3.82 (1.44)
** Symptom levels**	
Prolonged Grief levels, *M (SD)* (*n* = 225)	29.70 (9.54)
Insomnia levels, *M (SD)* (*n* = 219)	10.05 (7.43)
Posttraumatic stress levels, *M (SD)* (*n* = 221)	21.30 (15.97)
Depression levels, *M (SD)* (*n* = 224)	12.86 (9.29)
Grief rumination, *M (SD)* (*n* = 224)	36.46 (13.56)
Self-rated health levels, *M (SD)* (*n* = 223)	2.47 (1.04)
**Characteristics of the deceased children:**	
Child age (in years) when died, *M (SD)* (*n* = 151)	9.93 (6.54)
Child’s sex = female, *n* (%) (*n* = 151)	66 (43.7)
Duration of illness (in years), *M (SD)* (*n* = 151)	2.71 (3.51)
Relapse = yes, *n (%)* (*n* = 145)	66 (45.5)
** Diagnosis, ***n (%)* (*n* = 147)	
Brain tumors	59 (40.1)
Leukemia/lymphoma	45 (30.6)
Sarcoma	19 (12.9)
Other	24 (16.3)

### Model selection for latent classes of prolonged grief and insomnia symptoms

The fit indices of the models with one up to six classes are shown in Table 2. The fit indices did not clearly indicate the most optimal class solution. All entropy *R*^*2*^ values were ≥ 0.90 suggesting satisfactory class-separation. The BIC value was the lowest for the four-class solution, the SA-BIC was lowest for the five-class model, and the AIC went down when the number of classes increased. The significant (*p* < .05) BLRt indicated that the three-class model showed a better fit than the two-class model, which was not the case for the four-class model versus the three-class model. When taking the class sizes and interpretability of the three-class model into account, the three-class model was selected as the optimal model.

**Table 2:** Fit indices of the one- through six-class models (*N* = 225)

Model	LL	BIC(LL)	AIC(LL)	SABIC(LL)	Entropy *R*^2^	BLRt *p*-value	*N* smallest class
One-class model	-2526.04	5149.57	5088.08	5092.53			
Two-class model	-2058.04	4316.47	4190.07	4199.21	0.93	0.004	126/99
Three-class model	-1937.7	4178.71	3987.41	4001.23	0.91	0.028	92/75/58
Four-class model	-1864.88	4135.96	3879.76	3898.27	0.91	0.248	74/66/44/41
Five-class model	-1832.01	4173.12	3852.01	3875.22	0.91	0.422	68/62/42/37/16
Six-class model	-1803.28	4218.57	3832.55	3860.45	0.90	0.422	59/51/38/34/28/15

Note. AIC, Akaike Information Criterion; BIC, Bayesian Information Criterion; BLRt, Bootstrapped Likelihood Ratio test; LL = Loglikelihood; SA-BIC, sample size adjusted Bayesian Information Criterion

### Three latent classes of prolonged grief and insomnia symptoms

**Fig. 1:**
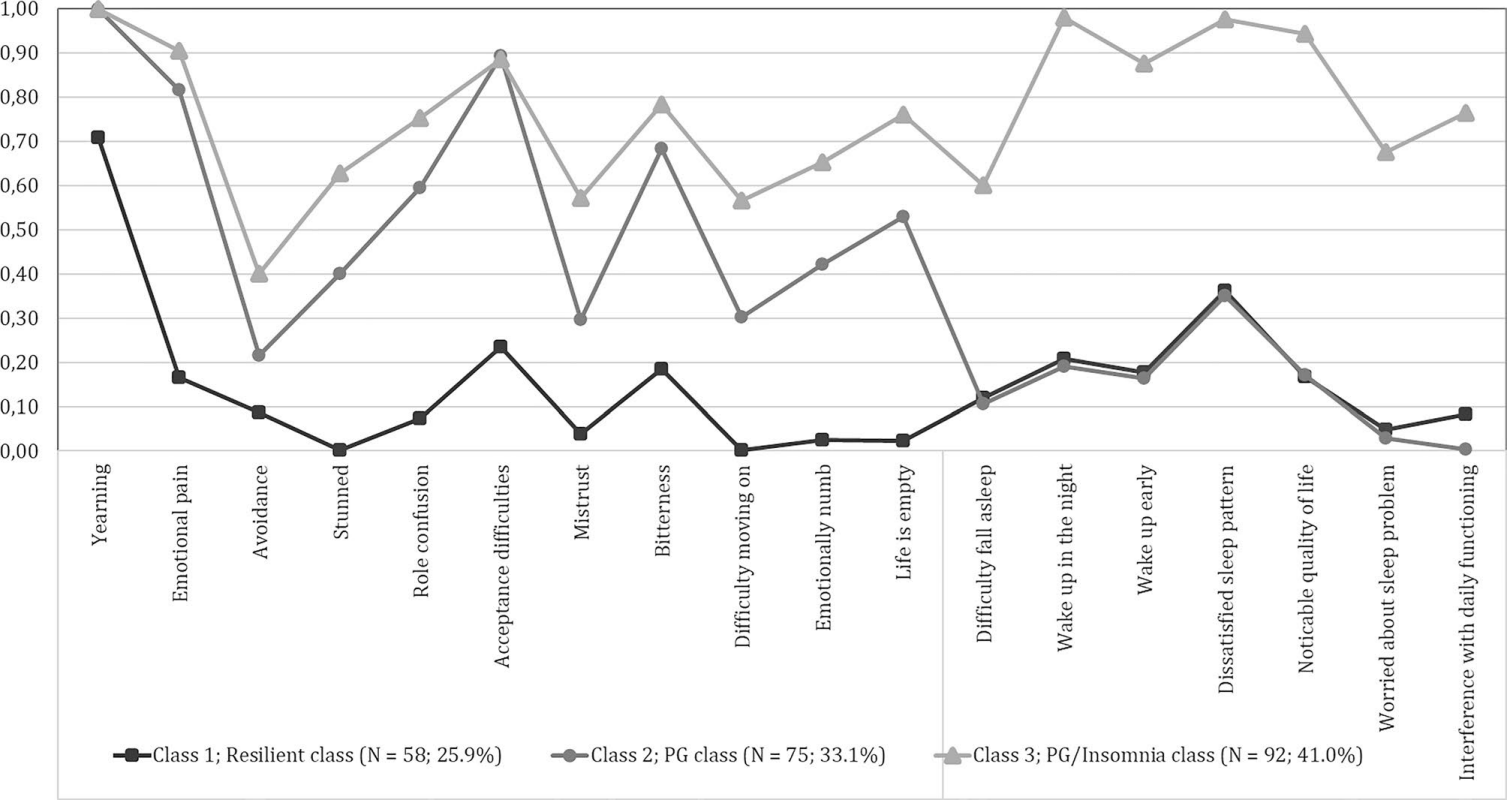
Probability estimates for the three-class model (*N* = 225)

The probability estimates of the three-class model are graphically displayed in Fig. 1 and shown in Table 3. Probability estimates of < 0.15 are considered low, estimates of ≥ 0.15 but ≤ 0.59 moderate, and estimates of ≥ 0.60 high, respectively [45]. The first class consisted of 58 persons (25.9%) who had a low to moderate probability of reporting prolonged grief and insomnia symptoms, except for the prolonged grief symptom “yearning”, which was reported by 71% of the persons in this class. This class was labelled as “resilient class”. About one third of the sample (*n* = 75, 33.1%) was allocated to a class that was characterized by moderate to high probability of reporting prolonged grief symptoms and low to moderate probability of reporting insomnia symptoms. This class was therefore named “prolonged grief class”. The largest class (*n* = 92, 41.0%) represented persons who had a high probability of reporting the majority of the prolonged grief and insomnia symptoms. We named this class the “prolonged grief/insomnia” class. Figures displaying the probability estimates for the other five class-models are displayed in Supplementary Figs. 1–5.

**Table 3:** Probability estimates for three-class model (*N* = 225)

	Symptom	Total (*n* = 225)		Resilient *n* = 58 (25.8%)		Prolonged Grief *n* = 75 (33.3%)		Prolonged Grief /Insomnia *n* = 92 (40.9%)	
		*N*	%	Est.	*SE*	Est.	*SE*	Est.	*SE*
Prolonged grief	Yearning	208	92.4	0.71	0.06	1.00	0.00	1.00	0.00
	Emotional pain	154	68.4	0.17	0.06	0.82	0.05	0.91	0.03
	Avoidance	58	25.8	0.09	0.04	0.21	0.05	0.40	0.05
	Stunned	88	39.1	0.00	0.01	0.40	0.06	0.63	0.05
	Role confusion	118	52.4	0.07	0.04	0.60	0.06	0.75	0.05
	Acceptance difficulties	162	72.0	0.23	0.07	0.89	0.04	0.89	0.03
	Mistrust	77	34.2	0.04	0.04	0.30	0.06	0.57	0.05
	Bitterness	134	59.6	0.18	0.04	0.68	0.06	0.78	0.04
	Difficulty moving on	75	33.3	0.00	0.01	0.30	0.06	0.57	0.05
	Emotionally numb	93	41.3	0.02	0.02	0.42	0.06	0.65	0.05
	Life is empty	111	49.3	0.02	0.02	0.53	0.06	0.76	0.05
Insomnia	Difficulty fall asleep	68	31.1	0.12	0.04	0.11	0.04	0.60	0.05
	Wake up in the night	113	51.6	0.21	0.06	0.19	0.05	0.98	0.02
	Wake up early	100	45.7	0.18	0.05	0.16	0.05	0.88	0.04
	Dissatisfied sleep pattern	133	60.7	0.36	0.07	0.35	0.06	0.98	0.02
	Noticeable quality of life	106	48.4	0.17	0.05	0.17	0.05	0.94	0.03
	Worried about sleep problem	65	29.7	0.05	0.03	0.03	0.02	0.68	0.05
	Interference with daily functioning	73	33.3	0.08	0.04	0.00	0.01	0.76	0.05

### Differences in symptomatology between the prolonged grief and insomnia classes

We examined, in separate models, whether the classes differed on levels of prolonged grief, insomnia, posttraumatic stress, depression, grief rumination, and self-rated health. Each class differed significantly, such that the resilient class showed lower levels on each of the six outcomes than the prolonged grief class. The prolonged grief class showed lower levels for each outcome than the prolonged grief/insomnia class. Results are shown in Table 4.

**Table 4:** Univariate associations with class-membership (*N* = 225)

	Resilient class *n* = 58 (25.8%)	Prolonged Grief class *n* = 75 (33.3%)	Prolonged Grief /insomnia class *n* = 92 (40.9%)	Class comparisons
**Symptoms levels**				
Prolonged Grief levels, *M (SD)* (*n* = 225)	18.10 (3.34)	30.63 (5.72)	36.25 (7.79)	1 < 2 < 3
Insomnia levels, *M (SD)* (*n* = 219)	5.06 (4.93)	4.93 (2.92)	17.44 (4.59)	1 = 2 < 3
Posttraumatic stress levels, *M (SD)* (*n* = 221)	7.02 (6.59)	18.99 (11.54)	32.39 (15.39)	1 < 2 < 3
Depression levels, *M (SD*) (*n* = 224)	4.93 (4.37)	10.71 (6.38)	19.69 (8.76)	1 < 2 < 3
Grief rumination, *M (SD)* (*n* = 224)	25.96 (8.40)	35.83 (10.97)	43.48 (13.79)	1 < 2 < 3
Self-rated health levels, *M (SD)* (*n* = 223)	1.71 (0.59)	2.28 (0.88)	3.11 (1.01)	1 < 2 < 3
**Sociodemographic correlates**				
Sex = female, *N (%)* (*n* = 225)	32 (55.2)	29 (52.0)	62 (67.4)	1 = 2; 1 = 3; 2 < 3;
Age (in years), *M (SD)* (*n* = 225)	44.10 (8.51)	45.22 (8.24)	47.89 (7.52)	1 = 2 < 3
Education = primary/secondary, *N (%)* (*n* = 224)	25 (43.1)	40 (53.3)	52 (57.1)	1 < 2; 1 = 3; 2 = 3
Employment = unemployed, *N (%)* (*n* = 224)	7 (12.1)	10 (13.3)	17 (18.7)	1 = 2 = 3
Marital status = single/living apart together, *N (%)* (*n* = 224)	11 (19.0)	4 (5.3)	26 (28.6)	1 > 2; 1 = 3; 2 < 3
**Loss-related correlates**				
Time since loss in years, *M (SD)* (*n* = 225)	3.91 (1.45)	3.51 (1.43)	4.02 (1.42)	1 = 2; 1 = 3; 2 < 3
Age (in years) of child at death, *M (SD)* (*n* = 225)	8.29 (6.72)	9.32 (6.52)	11.74 (6.18)	1 = 2 < 3
Only child died = yes, *N (%)* (*n* = 224)	3 (5.2)	4 (5.3)	16 (17.6)	1 = 2; 1 = 3; 2 < 3
Sickness duration (in years), *M (SD)* (*n* = 225)	2.29 (2.97)	3.91 (1.45)	2.98 (3.40)	1 = 2 = 3
Relapse of sickness = yes, *N (%)* (*n* = 216)	21 (37.5)	34 (47.2)	45 (51.1)	1 = 2 = 3

Note. All class comparisons that are indicated by < or > were significant at *p* < .05. Class comparisons that are indicated by = indicate non-significant difference, i.e., *p* ≥ .05

### Differences in terms of background- and loss-related characteristics between the classes

Each background- and loss-related characteristic was included as a correlate in the models separately (see Table 4). Persons in the prolonged grief/insomnia class were significantly more likely to be female than persons in the prolonged grief class. Those who belonged to the prolonged grief/insomnia class were significantly older than those in the resilient and prolonged grief class. Those in the prolonged grief class were more likely to be less educated than the resilient class. Significantly more persons were single/living apart together in the prolonged grief/insomnia and in the resilient class compared with the prolonged grief class. The loss occurred longer ago for persons in the prolonged grief/insomnia class than in the prolonged grief class. The age when the child died was also significantly older for persons in the prolonged grief/insomnia class than in the resilient and prolonged grief class. Lastly, the likelihood that persons in the prolonged grief/insomnia class lost their only child was greater than for persons in the prolonged grief class (see Supplementary Table 1 for details).

### Final model including all relevant correlates of prolonged grief and insomnia classes

When including the significant correlates (excluding prolonged grief and insomnia levels) simultaneously in one multivariate model (see Table 5), the findings indicate that age of the parent was still significantly related to class-membership such that persons in the prolonged grief class and persons in the prolonged grief/insomnia class were more likely to be older than the resilient class. Moreover, more persons were not married/living together in the resilient class compared with the prolonged grief class. Lastly, persons in the prolonged grief and prolonged grief insomnia classes reported significantly higher grief rumination levels compared with persons in the resilient class when taking all other relevant correlates into account.

**Table 5:** Correlates of class-membership when including relevant correlates simultaneously in one model (*N* = 218)

	B	SE (B)	95% confidence interval Class comparisons	
**Resilient class vs. Prolonged grief class**				
**Symptom levels**				
Posttraumatic stress levels	0.25	0.15	-0.04	0.54
Depression levels	-0.03	0.18	-0.38	0.31
Grief rumination	0.16	0.05	0.06	0.27
Self-rated health levels	0.34	0.41	-0.47	1.14
**Sociodemographic correlates**				
Sex = female	-0.57	0.76	-2.07	0.93
Age (in years)	0.21	0.08	0.05	0.38
Education = primary/secondary	1.76	1.02	-0.23	3.75
Marital status = single/living apart together	-3.27	1.16	-5.55	-0.99
**Loss-related correlates**				
Time since loss in years	-0.53	0.27	-1.06	0.00
Age (in years) of child at death	-0.11	0.13	-0.35	0.14
Only child died = yes	-0.07	1.06	-2.15	2.02
**Resilient class vs. Prolonged grief /Insomnia class**				
**Symptom levels**				
Posttraumatic stress levels	0.29	0.16	-0.02	0.60
Depression levels	0.07	0.18	-0.28	0.41
Grief rumination	0.18	0.06	0.07	0.29
Self-rated health levels	1.27	0.75	-0.20	2.75
**Sociodemographic correlates**				
Sex = female	0.25	0.95	-1.62	2.12
Age (in years)	0.30	0.12	0.06	0.54
Education = primary/secondary	1.24	1.08	-0.89	3.36
Marital status = single/living apart together	0.07	1.25	-2.37	2.51
**Loss-related correlates**				
Time since loss in years	-0.29	0.27	-0.82	0.23
Age (in years) of child at death	-0.10	0.14	-0.37	0.16
Only child died = yes	1.31	1.87	-2.36	4.99
**Prolonged grief class vs. Prolonged grief/Insomnia class**				
**Symptom levels**				
Posttraumatic stress levels	0.04	0.05	-0.06	0.14
Depression levels	0.10	0.05	-0.01	0.21
Grief rumination	0.02	0.03	-0.03	0.07
Self-rated health levels	0.94	0.77	-0.58	2.45
**Sociodemographic correlates**				
Sex = female	0.82	0.78	-0.70	2.34
Age (in years)	0.08	0.08	-0.07	0.24
Education = primary/secondary	-0.52	0.71	-1.91	0.87
Marital status = single/living apart together	3.34	1.84	-0.26	6.94
**Loss-related correlates**				
Time since loss in years	0.24	0.19	-0.14	0.61
Age (in years) of child at death	0.00	0.06	-0.11	0.11
Only child died = yes	1.38	1.78	-2.11	4.87

## Discussion

The primary aim of this study was to identify profiles of insomnia and prolonged grief symptoms in parents bereaved of a child to cancer. Comparing multiple LCA models, a three-class solution was found to be the most optimal. We identified a resilient class (25.9%), with predominantly low to moderate odds of insomnia and prolonged grief symptoms, a prolonged grief class (33.1%), with moderate to high probability of prolonged grief symptoms and low to moderate probability of insomnia symptoms, and a comorbid prolonged grief/insomnia class (41.0%), with predominantly high odds of prolonged grief and insomnia symptoms.

As a secondary aim, we sought to explore the correlates of class membership. Univariate analyses demonstrated that those in the comorbid class showed higher prolonged grief, posttraumatic stress, and depression symptom levels, and grief rumination than those in the prolonged grief class. The prolonged grief class, in turn, showed higher levels of these variables than the resilient class. Insomnia symptoms were higher in the comorbid class relative to the other two classes. Self-rated health was also relatively lower in the classes showing the more severe symptomatology. A variety of sociodemographic and loss-related variables related to class membership. Notably, persons were more likely to be part of the insomnia/prolonged grief class than the other classes if they were older, female, and married or living together. A longer time since loss, older age of the child at death, and the death of an only child was also accompanied by higher odds of being part of the comorbid class relative to the other classes. In a multivariate analysis, including all covariates simultaneously, only grief rumination and older age of the child at death predicted higher odds of membership of the prolonged grief/insomnia class relative to the resilient class, and the prolonged grief class relative to the resilient class.

The three-class solution showed that a large proportion of parents had moderate to high prolonged grief symptoms, with nearly three quarters belonging to classes with elevated symptoms, in line with previous studies showing elevated symptomatology among bereaved parents [8, 9, 46]. Furthermore, two-fifths of the sample additionally showed high odds of co-occurring insomnia symptoms. What this study adds to the existing literature is that we now showed that a subgroup of bereaved people experiences co-occurring prolonged grief and insomnia symptoms, underscoring meaningful heterogeneity in post-bereavement adjustment. This finding aligns with previous research showing positive concurrent and longitudinal associations between prolonged grief and sleep disturbances [4, 6, 7, 17, 18]. Moreover, it complements a recent trajectory study demonstrating that two-thirds of bereaved persons who develop chronic insomnia have a probable PGD [19]. Clinically, this suggests that screening for both sleep and grief symptoms may help identify parents in need of targeted support and monitoring.

A third of the sample presented with high odds of prolonged grief with low to moderate odds of insomnia symptoms. This suggests that prolonged grief symptomatology can be discerned from symptoms of related disorders, which corresponds with findings from other LCAs and factor analytic studies [e.g., 26, 47, 48], demonstrating that prolonged grief constitutes a unique construct. Only a quarter of the sample demonstrated resilient responses, characterized by low to moderate odds of endorsing prolonged grief and insomnia symptoms. As resilience is generally the modal response to major stressful life-events, occurring in about two-thirds of persons exposed to them [49], this illustrates the unique vulnerability of bereaved parents to the development of mental health problems [9, 31, 50, 51].

The univariate analyses of covariates of class membership yielded a number of notable findings. First, these analyses showed that symptom levels of depression and posttraumatic stress were higher in the classes with the higher odds of endorsing prolonged grief and/or insomnia symptoms. This aligns with prior research demonstrating the frequent co-occurrence of severe prolonged grief, posttraumatic stress, and depressive symptoms [for a review: 27], as well as research demonstrating that comorbidity, particularly comorbid depression, heightens the odds of sleep disturbances in persons with probable prolonged grief [4, 6, 7]. Grief rumination followed a similar pattern as depressive and posttraumatic stress symptomatology, which corresponds with the past research demonstrating positive concurrent and longitudinal associations of rumination with prolonged grief and insomnia symptoms [28, 29, 52–55]. Self-rated health was lower for the classes with the higher odds of endorsing prolonged grief and/or insomnia symptoms. This stands to reason, considering the range of physical health problems linked with prolonged grief severity, such as cardiovascular health problems [56] and immunological deficiencies [57], and with insomnia severity, such as changes in metabolism and elevated cortisol levels [58].

When looking at sociodemographic characteristics, it is notable that older persons and women were relatively more likely to be in the prolonged grief/insomnia class, which may imply that these persons are at heightened risk for these comorbid mental health problems. However, when interpreting these findings, it should be considered that old age and female gender are also risk factors of sleep disturbances in the general population [4, 58]. Some loss-characteristics also related to class membership. The loss of an older child and the loss of an only child were also more common among persons in the prolonged grief/insomnia class. The first finding suggests that a longer relationship with a child may elicit more severe grief, possibly due to the stronger attachment bonds that may have been formed over the years [59]. The second finding aligns with prior research in Chinese parents who lost an only child, termed Shidu parents, who appear at risk of severe mental health problems, including insomnia and prolonged grief [60, 61]. Possibly, the presence of remaining children can provide a parent with an ongoing family life offering comfort and support, which could act as a protective factor in psychological adaptation to loss. Some correlates of class membership were more difficult to interpret. For example, parents who were married or cohabiting were most common in the prolonged grief class relative to the other classes. A longer time since loss was related to relatively higher odds of being in the prolonged grief/insomnia class. This seems counterintuitive, as post-loss prolonged grief and insomnia symptoms generally decline over time [e.g., 18]. Nevertheless, parents bereaved of their child often struggle for many years after the loss [9, 50, 51, 62]. It could be that it is a sampling issue; voluntary response sampling may have led to an overrepresentation of persons severely affected by the death several years post-loss.

Multivariate analyses of covariates of class membership helped identify the strongest correlates of class membership. Older age, as a general risk factor for sleep problems, consistently distinguished the prolonged grief/insomnia class from the other two classes. More interesting from a clinical perspective was that grief rumination was significantly higher in the prolonged grief/insomnia class relative to the other two classes. As mentioned, this corresponds with prior empirical research. This corresponds with the cognitive model of insomnia by Harvey [63], who proposed that repetitive thinking leads to arousal and distress, which in turn elicits selective attention and increased monitoring of sleep-related threats. This selective attention results in overestimation of sleep impairment and daytime consequences, which fuels repetitive thinking about sleep and increases physiological arousal, causing impairment in sleep. The finding can also be reconciled with theories about rumination in bereavement. For example, the Rumination as Avoidance Hypothesis holds that rumination on the causes and consequences of the loss could serve to suppress/distract oneself from painful aspects of the loss, thereby hampering the cognitive processing of the loss and exacerbating grief [for a review: 64]. Clinically, findings support the use of therapeutic strategies focused on the reduction of rumination in bereaved persons with comorbid insomnia and prolonged grief. An example of such a strategy includes exposure to avoided aspects of the loss, proven effective in reducing rumination and prolonged grief [65]. Another key technique, applied in CBT-I [66], is cognitive restructuring of maladaptive sleep-related thoughts and cognitions. CBT-I has yielded prolonged grief and insomnia symptom reductions in a recent small randomized controlled trial [20].

### Strengths, limitations and future research directions

A clear strength of the study is the inclusion of a large national sample of persons at risk of experiencing post-loss mental health problems, i.e., parents bereaved of a child. Despite the unicity and strengths of our approach, some limitations warrant consideration. First, our study focused exclusively on parents who lost a child to cancer, and grief trajectories may differ for parents bereaved due to other causes such as sudden or accidental deaths. Therefore, caution is warranted when generalizing these findings beyond cancer-related bereavement. Second, despite a registry-based sampling approach, the present sample is still a voluntary response sample, which has led to a slight overrepresentation of women. Future research should establish whether results generalize to samples including more men. This appears particularly pertinent as the variable related to class membership in the univariate analyses of covariates. Third, the PG-13, used to assess prolonged grief symptoms in this study, does not comprehensively assess symptoms of PGD as included in the ICD-11 and DSM-5-TR [67]. Future research should use instruments designed to capture the symptoms of these new diagnoses, such as the Traumatic Grief Inventory Self Report Plus [68]. Moreover, we could not assess actual caseness of insomnia and PGD within the identified classes: clinical interviews would be required and are recommended for future research.

## Conclusions

Based on this LCA study, there appear to be three classes of bereaved parents: a prolonged grief/insomnia class, a prolonged grief class, and a resilient class. Overall, the study confirmed that many bereaved parents experience severe and persistent grief and that such grief reactions often co-occur with sleep disturbances. Resilient responses were relatively rare. Subsequent multivariate analyses of covariates of class membership demonstrated that older age heightens the risk of co-occurring prolonged grief and insomnia symptomatology. Moreover, grief rumination emerged as a unique predictor of class membership, supporting continued scientific and clinical attention for repetitive thought in the treatment of prolonged grief and insomnia in bereaved adults.

## Supplementary Information

Below is the link to the electronic supplementary material.

Supplementary Material 1

Supplementary Material 2

Supplementary Material 3

Supplementary Material 4

Supplementary Material 5

Supplementary Material 6

## Data Availability

All data generated or analysed during this study are not publicly available due the restrictions from the ethics committee. Reasonable requests can be addressed to the corresponding author.

## Abbreviations

PGD: Prolonged grief disorder
PTSD: Posttraumatic stress disorder
ICD-11: International Classification of Diseases, eleventh edition
DSM-5-TR: The Diagnostic and Statistical Manual of Mental Disorders, fifth edition, text revision
CBT-I: Cognitive-behaviour therapy for insomnia
LCA: Latent class analysis
PG-13: Prolonged Grief Disorder-13
ISI: Insomnia Severity Index
UGRS: Utrecht Grief Rumination Scale
PCL-5: Posttraumatic Stress Disorder Checklist for DSM-5
MADRS: Montgomery-Åsberg Depression Rating Scale
SRH: Self-rated health
AIC: Akaike’s Information Criterion
BIC: Bayesian Information Criterion
SA-BIC: Sample-Size Adjusted Bayesian Information Criterion
BLRt: The bootstrap likelihood ratio test
CI: Confidence interval
