# Supplementary material for: Prolonged grief and insomnia symptoms in cancer-bereaved parents: a latent class analysis
Source: BMC Psychiatry. 2026 Mar 13;26:288. doi: 10.1186/s12888-026-07955-9 (PMC13063547; doi:10.1186/s12888-026-07955-9)
Supplement: Supplementary file 3 — Supplementary Material 3 [file 12888_2026_7955_MOESM3_ESM.docx]

**Supplementary Figure 3**. Plot probability estimates 4-class model
