# Supplementary material for: Prolonged grief and insomnia symptoms in cancer-bereaved parents: a latent class analysis
Source: BMC Psychiatry. 2026 Mar 13;26:288. doi: 10.1186/s12888-026-07955-9 (PMC13063547; doi:10.1186/s12888-026-07955-9)
Supplement: Supplementary file 6 — Supplementary Material 6 [file 12888_2026_7955_MOESM6_ESM.docx]

**Supplementary Table 1**

Univariate associations with class-membership

|  | B | *SE* (B) | 95% confidence interval | |
| --- | --- | --- | --- | --- |
| Resilient class vs. Prolonged grief class |  |  |  |  |
| Symptoms levels |  |  |  |  |
| Prolonged grief levels (*n* = 225) | 0.78 | 0.12 | 0.54 | 1.02 |
| Insomnia levels (*n* = 219) | 0.03 | 0.07 | -0.10 | 0.16 |
| Posttraumatic stress levels (*n* = 221) | 0.25 | 0.06 | 0.13 | 0.37 |
| Depression levels (*n* = 224) | 0.24 | 0.05 | 0.14 | 0.34 |
| Grief rumination (*n* = 224) | 0.19 | 0.04 | 0.11 | 0.28 |
| Self-rated health levels (*n* = 223) | 0.08 | 0.02 | 0.05 | 0.11 |
| Sociodemographic correlates |  |  |  |  |
| Sex = women (*n* = 225) | -0.20 | 0.38 | -0.94 | 0.54 |
| Age (in years) (*n* = 225) | 0.02 | 0.03 | -0.04 | 0.07 |
| Education = lower/secondary school (*n* = 224) | 0.06 | 0.02 | 0.02 | 0.11 |
| Employment = unemployed (*n* = 224) | 0.15 | 0.58 | -0.98 | 1.28 |
| Marital status = not married/living together (*n* = 224) | -1.78 | 0.79 | -3.33 | -0.22 |
| Loss-related correlates |  |  |  |  |
| Time since loss in years (*n* = 225) | -0.26 | 0.13 | -0.52 | 0.01 |
| Age (in years) when child died (*n* = 225) | -0.26 | 0.13 | -0.52 | 0.01 |
| Only child died = yes (*n* = 224) | 0.15 | 0.88 | -1.57 | 1.88 |
| Sickness duration (in years) (*n* = 225) | 0.02 | 0.07 | -0.12 | 0.16 |
| Sickness came back after recovery = yes (*n* = 216) | 0.32 | 0.39 | -0.44 | 1.08 |
| Resilient class vs. Prolonged grief/Insomnia class |  |  |  |  |
| Symptoms levels |  |  |  |  |
| Prolonged grief levels (*n* = 225) | 0.78 | 0.12 | 0.54 | 1.02 |
| Insomnia levels (*n* = 219) | 0.69 | 0.15 | 0.41 | 0.98 |
| Posttraumatic stress levels (*n* = 221) | 0.25 | 0.06 | 0.13 | 0.37 |
| Depression levels (*n* = 224) | 0.42 | 0.06 | 0.31 | 0.54 |
| Grief rumination (*n* = 224) | 0.24 | 0.04 | 0.15 | 0.33 |
| Self-rated health levels (*n* = 223) | 2.41 | 0.37 | 1.67 | 3.14 |
| Sociodemographic correlates |  |  |  |  |
| Sex = women (*n* = 225) | 0.51 | 0.36 | -0.19 | 1.20 |
| Age (in years) (*n* = 225) | 0.06 | 0.02 | 0.02 | 0.11 |
| Education = lower/secondary school (*n* = 224) | 0.51 | 0.35 | -0.17 | 1.20 |
| Employment = unemployed (*n* = 224) | 0.15 | 0.58 | -0.98 | 1.28 |
| Marital status = not married/living together (*n* = 224) | 0.52 | 0.42 | -0.30 | 1.33 |
| Loss-related correlates |  |  |  |  |
| Time since loss in years (*n* = 225) | 0.03 | 0.12 | -0.21 | 0.27 |
| Age (in years) when child died (*n* = 225) | 0.09 | 0.03 | 0.03 | 0.15 |
| Only child died = yes (*n* = 224) | 1.38 | 0.71 | -0.02 | 2.77 |
| Sickness duration (in years) (*n* = 225) | 0.06 | 0.05 | -0.05 | 0.17 |
| Sickness came back after recovery = yes (*n* = 216) | 0.53 | 0.36 | -0.17 | 1.24 |
| Prolonged grief class vs. Prolonged grief/Insomnia class |  |  |  |  |
| Symptoms levels |  |  |  |  |
| Prolonged grief levels (*n* = 225) | 0.13 | 0.03 | 0.08 | 0.19 |
| Insomnia levels (*n* = 219) | 0.67 | 0.10 | 0.47 | 0.86 |
| Posttraumatic stress levels (*n* = 221) | 0.08 | 0.02 | 0.05 | 0.11 |
| Depression levels (*n* = 224) | 0.18 | 0.03 | 0.12 | 0.24 |
| Grief rumination (*n* = 224) | 0.05 | 0.01 | 0.02 | 0.07 |
| Self-rated health levels (*n* = 223) | 2.41 | 0.37 | 1.67 | 3.14 |
| Sociodemographic correlates |  |  |  |  |
| Sex = women (*n* = 225) | 0.71 | 0.34 | 0.05 | 1.37 |
| Age (in years) (*n* = 225) | 0.06 | 0.02 | 0.02 | 0.11 |
| Education = lower/secondary school (*n* = 224) | 0.23 | 0.33 | -0.42 | 0.87 |
| Employment = unemployed (*n* = 224) | 0.44 | 0.45 | -0.45 | 1.32 |
| Marital status = not married/living together (*n* = 224) | 2.30 | 0.74 | 0.85 | 3.74 |
| Loss-related correlates |  |  |  |  |
| Time since loss in years (*n* = 225) | 0.29 | 0.12 | 0.05 | 0.52 |
| Age (in years) when child died (*n* = 225) | 0.07 | 0.03 | 0.02 | 0.12 |
| Only child died = yes (*n* = 224) | 1.23 | 0.60 | 0.04 | 2.41 |
| Sickness duration (in years) (*n* = 225) | 0.04 | 0.06 | -0.07 | 0.15 |
| Sickness came back after recovery = yes (*n* = 216) | 0.21 | 0.33 | -0.44 | 0.87 |
